# Zosyn-Induced Rapid Thrombocytopenia in a Patient With End-Stage Renal Disease and HIV: A Case Report

**DOI:** 10.7759/cureus.50167

**Published:** 2023-12-08

**Authors:** Santosh Adhikari, Patrick Buoniconti, Mounika Lakshmi Allam

**Affiliations:** 1 Internal Medicine, MacNeal Hospital, Berwyn, USA

**Keywords:** rapid thrombocytopenia, thrombocytopenia, tazobactum, piperacillin, zosyn

## Abstract

Thrombocytopenia is a rare but potentially serious complication associated with the use of various medications, including antibiotics. Piperacillin-tazobactam (Zosyn), a commonly used broad-spectrum antibiotic, has been reported as an infrequent cause of drug-induced thrombocytopenia. We present a case of a 65-year-old female with end-stage renal disease (ESRD) on hemodialysis, HIV, and multiple comorbidities who developed rapid-onset thrombocytopenia shortly after receiving Zosyn. The patient's platelet count dropped from a baseline of 291,000/μL on admission to a nadir of 8,000/μL within 36 hours of starting Zosyn. The administration of Zosyn was promptly discontinued, and the patient's platelet count gradually increased to 134,000/μL within two days after discontinuation. The patient had no apparent bleeding manifestations during her hospital stay. Further workup for other causes of thrombocytopenia, including heparin-induced thrombocytopenia (HIT), was negative. This case highlights the importance of vigilance for drug-induced thrombocytopenia in patients receiving Zosyn and the need for prompt recognition and management to prevent potential complications.

## Introduction

Thrombocytopenia is a relatively uncommon but critical adverse effect of various medications [1]. Drug-induced immune-mediated thrombocytopenia causes a more rapid decline in platelet count than myelosuppression [2]. Piperacillin-tazobactam (Zosyn), a widely used antibiotic, has been rarely associated with acute and severe drug-induced thrombocytopenia [3]. Here, we present a case of Zosyn-induced thrombocytopenia in a 65-year-old female with end-stage renal disease (ESRD) and HIV.

## Case presentation

The patient is a 65-year-old female with a medical history of ESRD on hemodialysis, HIV, hypertension, hyperlipidemia, ischemic cardiomyopathy with questionable congestive heart failure, peripheral vascular disease, type 2 diabetes, prior cerebrovascular accident (CVA) with speech difficulties and cognitive slowing, and chronic anemia. She was admitted to the ED for evaluation of possible anemia but was found to be lethargic and hypothermic. Her blood pressure was noted to be systolic (in the range of 220 mmHg and above), and she was hypoglycemic on admission, which was corrected with dextrose. The patient was placed on a bear hugger for hypothermia. Given the hypothermia and a heart rate of 95 beats per minute, sepsis was considered in the differential diagnosis. A chest X-ray revealed right-sided pleural effusion with consolidation and fluid in the fissure, likely secondary to ESRD with a possible component of heart failure.

**Figure 1:**
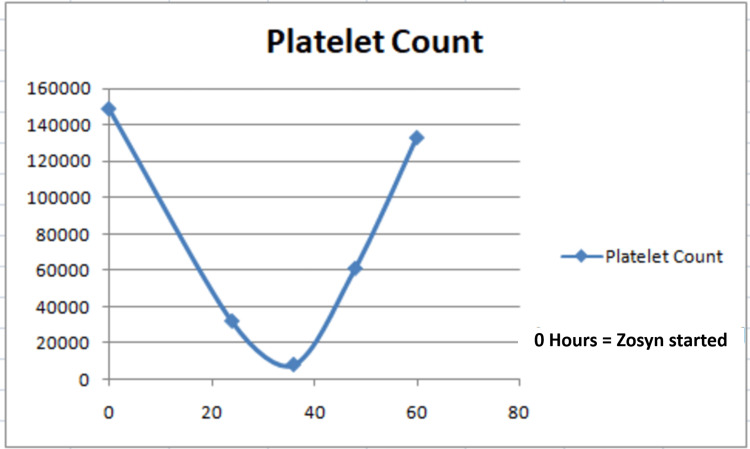
Platelet trend from zero hours to the lowest point following Zosyn (piperacillin-tazobactam) administration Zosyn was discontinued after the nadir

The patient was started on Zosyn. On admission, her platelet count was 291,000/μL, which gradually dropped to 8,000/μL, approximately 36 hours after Zosyn initiation. Concerns about drug-induced thrombocytopenia prompted the utilization of the Naranjo adverse event probability scale [4], resulting in a score of eight, indicative of a probable adverse drug reaction. After the discontinuation of Zosyn, her platelet counts gradually increased to 134,000/μL within two days. Throughout her hospital stay, the patient did not exhibit any apparent bleeding manifestations. Lactate dehydrogenase was normal, with a peripheral smear revealing normal shapes of scant platelets. Vitamin B12 and folate were found to be normal. Zosyn was discontinued and the patient was transitioned to vancomycin. The platelet counts returned to normal after the transition to vancomycin, as shown in Figure 1.

Furthermore, extensive diagnostic investigations were performed to rule out alternative causes of thrombocytopenia, such as heparin-induced thrombocytopenia (HIT), disseminated intravascular coagulation (DIC), and immune thrombocytopenic purpura (ITP), as shown in Table 1.

**Table 1:** Thrombocytopenia workup

Work up	Patient’s values with normal range
Fibrinogen	387 mg/dl (200-400 mg/dl)
Lactate dehydrogenase	205 U/L (108-212 U/L)
Haptoglobin	39 mg/dl (36-195 mg/dl)
Platelet factor 4 antibody	Negative
Vitamin B12	398 pg/ml (180-914 pg/ml)

Notably, these conditions were not supported by clinical findings or laboratory results.

## Discussion

Drug-induced immune thrombocytopenia is a recognized condition that is sometimes neglected, often overshadowed by concerns about other potentially life-threatening causes. Generally, thrombocytopenia is categorized based on its underlying causes, which may involve reduced production, heightened destruction, dilution, or sequestration. The potential severity of thrombotic microangiopathies and heparin-induced thrombocytopenia often leads to their exclusion during the initial assessment of patients with low platelet counts due to the associated high mortality and morbidity. Additionally, consideration is given to immune thrombocytopenia and malignant bone marrow conditions when investigating cases of thrombocytopenia [2].

The rapid-onset thrombocytopenia observed in this case shortly after Zosyn (piperacillin-tazobactam) administration raises suspicion for drug-induced immune-mediated thrombocytopenia [3,5]. The timeline of events, with the patient's platelet count decreasing shortly after the initiation of Zosyn and returning to baseline upon its discontinuation, strongly supports this suspicion [6].

The Naranjo adverse event probability scale was employed to assess the likelihood of an adverse drug reaction [4]. With a calculated score of eight, this scale suggests a probable adverse drug reaction in this case, further reinforcing the association between Zosyn and thrombocytopenia.

In conclusion, this case underscores the importance of considering drug-induced thrombocytopenia when faced with unexplained thrombocytopenia in a clinical setting. The rapid onset and resolution of thrombocytopenia upon Zosyn discontinuation, coupled with the Naranjo scale assessment [4], strongly suggest a causal relationship. Drug-induced thrombocytopenia can be overlooked, especially in patients with several comorbidities [7, 8]. Whenever severe thrombocytopenia occurs, it is important to consider the differential diagnosis of drug-induced thrombocytopenia. Clinicians should be vigilant for such adverse reactions, especially when using drugs known to be associated with hematologic complications.

## Conclusions

This case highlights the occurrence of Zosyn-induced thrombocytopenia in a 65-year-old female with ESRD who was on hemodialysis and had HIV. Prompt recognition and discontinuation of Zosyn led to the resolution of thrombocytopenia without apparent bleeding manifestations. Healthcare providers should remain vigilant for drug-induced thrombocytopenia in patients receiving Zosyn, particularly those with underlying comorbidities. The Naranjo scale helps establish an association between drug administration and thrombocytopenia, guiding timely management and preventing potential complications.
